# Peptides and Peptidomimetics for Antimicrobial Drug Design

**DOI:** 10.3390/ph8030366

**Published:** 2015-07-13

**Authors:** Biljana Mojsoska, Håvard Jenssen

**Affiliations:** Department of Science, Systems and Models, Roskilde University, Universitetsvej 1, Postboks 260, Roskilde 4000, Denmark

**Keywords:** antimicrobial peptides, mechanism of action, peptidomimetics

## Abstract

The purpose of this paper is to introduce and highlight a few classes of traditional antimicrobial peptides with a focus on structure-activity relationship studies. After first dissecting the important physiochemical properties that influence the antimicrobial and toxic properties of antimicrobial peptides, the contributions of individual amino acids with respect to the peptides antibacterial properties are presented. A brief discussion of the mechanisms of action of different antimicrobials as well as the development of bacterial resistance towards antimicrobial peptides follows. Finally, current efforts on novel design strategies and peptidomimetics are introduced to illustrate the importance of antimicrobial peptide research in the development of future antibiotics.

## 1. Introduction

**Figure 1:**
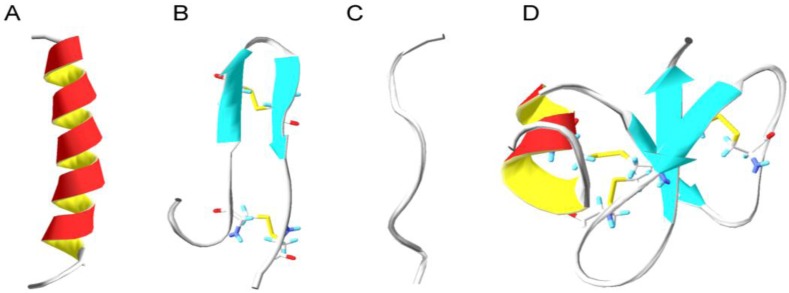
Four structural classes of antimicrobial peptides. (**A**) α-helical structure of human cathelicidin LL-37 (PDB code 2K6O [4]); (**B**) β-sheeted polyphemusin (PDB code 1RKK [5]); (**C**) extended indolicidin (PDB code 1G89 [6]); (**D**) and mixed structures like human β-defensin-2 (PDB code 1FQQ [7]).

Even though humans have adapted to live in harmony with different microorganisms throughout evolution, this balanced symbiotic relationship can sometimes shift and allow pathogenic bacteria to blossom and cause infections. In the struggle for survival, a complex mechanism involving many key components assists in the elimination of these infectious agents. Antimicrobial peptides are conserved biomolecules among all living species, including bacteria that take part in the battle against the invading pathogens. They are relatively short (<100 amino acid residues), positively charged, amphipathic (have both hydrophobic and hydrophilic domains) and exhibit diversity based on their structural properties [1]. Despite the structural difference and myriad of sequences incorporating both natural and unnatural amino acids, antimicrobial peptides exhibit broad spectrum antibacterial activities. The ability of antimicrobial peptides to kill or inhibit the growth of bacteria has attracted the attention of many research groups worldwide to study mechanism(s) of their antimicrobial action. Their amphipathic nature is believed to allow them to interact with the negatively charged structures and hydrophobic fatty acid chains found on the target microbial membranes, leading to membrane destabilization and apparently cell lysis [2]. Generally AMPs are classified in four large families based on their secondary conformations in α-helices, β-sheets, mixed structures and non- α- or β-structures (extended) [3] (Figure 1).

These structures are predominantly present upon interaction with lipid membranes. Most antimicrobial peptides from both multi- and unicellular organisms are derived from precursor sequences. Consequently, they display a number of post-translational modifications that largely modify their activity. These include: proteolytic processing, glycosylation, amidation, halogenation, phosphorylation, incorporation of unnatural d-amino acids and cyclization [8]. Some AMPs are also synthesized non-ribosomely e.g., gramicidin S and lipopeptides. Antimicrobial peptides have also been intensively modified through synthetic chemistry in order to meet the requirements of potential therapeutic drugs, thus further increasing the structural diversity. Most recently, combinations of different structures such as β-peptides, peptoids, β-peptoids, peptide-peptoid hybrids and other, have been synthesized and the antimicrobial activity of the resulting peptidomimetics has been compared to that of traditional antimicrobial peptides. This extensive research holds vast potential in dissecting the detailed mechanism by which both innate and novel antimicrobial compounds can be used to fight pathogenic infections. In addition, there is a remarkable interest for development of new antimicrobials to combat infectious diseases in parallel with the increasing evidence of their broad spectrum activity. Until now more than 2000 antimicrobial peptides (also referred to as host defense peptides) have been isolated from various cells and tissues of animals, insects, plants and bacteria (http://aps.unmc.edu/AP/main.php) [9]. Well known examples of antimicrobial peptides belong to the families of the cathelicidins and defensins (found in many insects and plants and animals, including humans), thionins (isolated from plants), cecropins (found in the hemolymph of the cecropia silk moth in the early 80s) and magainins (secreted from frog skin) [10,11]. This review will focus on these peptide groups, discuss their antibacterial mode of action in parallel with their structural characteristics, and then further elute to the potential of peptidomimetic as novel antimicrobial agents and how they can be designed based on our current knowledge on antimicrobial peptides.

## 2. Antimicrobial Peptides Isolated from Mammals

In mammals, antimicrobial peptides have been isolated from different sources such as the granules of neutrophils, Peneth cells, mucosal secretions from epithelial cells and as protein degradation products [12]. Three classes of antimicrobial peptides, found in abundance in neutrophils defensins, cathelicidins and histatins [13,14,15] have been studied extensively by several groups. This section highlights some important research on defensins and cathelicidins.

### 2.1. Defensins

**Figure 2:**
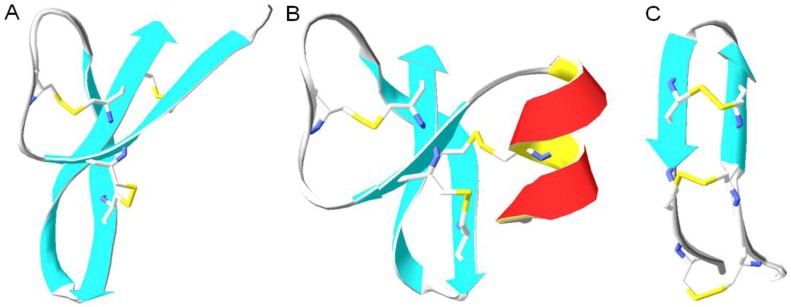
Three structural classes of defensines. The PDB codes are all indicated, in addition to the primary sequence with the disulfide bridges indicated with numbers in subscript (**A**) Human alpha-defensin-6 (PDB code 1ZMQ) AFTC_1_HC_2_RRSC_3_YSTEYSYGTC_2_TVMGINHRFC_3_C_1_L [31]; (**B**) Human beta-defensin-1 (PDB code 1IJV) DHYNC_1_VSSGGQC_2_LYSAC_3_PIFTKIQGTC_2_YRGKAKC_1_C_3_K [32] and (**C**) rhesus theta defensin-1 (PDB code 1HVZ) GFC_1_RC_2_LC_3_RRGVC_3_RC_2_IC_1_TR [33].

Defensins, first discovered in human neutrophils, are 18-45 amino acids long cationic molecules which are isolated from mast cells and tissues involved in host defense [16,17,18]. They have been classified as α-, β and θ-defensins based on structural differences (Figure 2). All three classes are rich in cysteine and arginine residues. The difference between the classes lies in the position of disulfide linked cysteine residues. Herein, the first class of defensins (α-defensins), have disulfide connections between cysteine residues 1-6, 2- and 3-5, whereas β-defensins disulfide bridges are located between cysteine residues 1-5, 2-4 and 3-6 (Table 1) [19]. Moreover, human α-defensins are less cationic, shorter and more hydrophobic than human β-defensins [20]. Human neutrophil peptides (HNP1-HNP4) and human defensins (HD-5 and HD-6) are six different human α-defensins found in monocytes, NK cells, B and T cells, neutrophils and Peneth cells, respectively [21,22]. Studies show that HNPs exhibit antimicrobial activity against both Gram-negative and Gram-positive bacteria [23]. Human defensin 5 exerts its antimicrobial activity against various bacteria such as *Escherichia coli*, *Listeria monocytogenes*, *Salmonella typhimurium*, *Staphylococcus aureus* and *Vibrio cholerae* [24]. Contrary to α-defensins, four human β-defensins (HBD 1-4) have been isolated from leukocytes and epithelial cells. The β-defensins also exhibit microbicidal activity against a panel of bacteria, with HBD-4 being the most potent against *Pseudomonas aeruginosa* [25]. It has been shown that epithelial human β-defensins are over-expressed in patients suffering from psoriasis, a chronic skin inflammation, with characteristic skin lesions cleared of infection. Conversely in atopic dermatitis where the expression of HBDs is suppressed, the lesions are infection-prone [26], clearly illustrating the importance of these antimicrobial peptides in host defense.

### 2.2. Cathelicidins

The cathelicidins are another class of antimicrobial peptides which have been identified in many vertebrates such as fish, bird, cow, pig, rabbit, sheep, mouse, monkey, horse and human [27,28,29]. They are primarily produced in epithelial cells, neutrophils and macrophages. Indolicidin and human cathelicidin LL-37 (Table 1) are two of the most studied members of this class of peptides. LL-37 is the only member of this group of peptides found in human. The importance of these peptides is elucidated in studies where increased susceptibility to infections is observed in patients deficient in neutrophil production of LL-37, the major source of human antimicrobial peptides [30].

Indolicidin is a small antimicrobial peptide with 13 amino acids isolated from bovine neutrophils (Figure 1 and Table 1). It is rich in tryptophan (39%) and arginine (23%) residues, and it is amidated at the *C*-terminal arginine [34,35]. The antimicrobial activity towards both Gram-negative and Gram-positive bacteria, fungi and protozoa is most likely not a result of any confined secondary structure, as nuclear magnetic resonance spectroscopy studies failed to report defined secondary or amphipathic structure which is characteristic for majority of antimicrobial peptides [6,36]. However the hydrophobic and positively charged domains of indolicidin are crucial for its interactions with bacterial pathogens. In terms of mechanistic studies, it has been shown that indolicidin failed to cause bacterial lysis even at concentrations up to 4 times the MIC concentration, thus excluding traditional membrane depolarization and pore models. In addition, indolicidin is capable of binding to DNA and inhibiting DNA synthesis which results in cell filamentation [36,37,38]. Recent studies have also demonstrated that the inhibition of DNA replication and transcription is due to the peptides central PWWP motif, which is able to wrap around and stabilize DNA structures [39]. In order to improve the potency of indolicidin, the effect of various structural modifications has been tested. These include tryptophan replacement with non-natural amino acids [40], expression of mutant structures such as CP10A (ILAWKWAWWAWRR-NH2) and CP11 (ILKKWPWWPWRRK-NH2) with increased potency against Gram-positive bacteria [41] and enhanced cationicity which improved the hemolytic profile and the activity against Gram-negative bacteria [42]. Additionally, studies have shown that cyclization of indolicidin improved the peptide proteolytic stability without significantly affecting the peptide antibacterial mode of action and activity [43].

**Table 1:** Overview of some natural antimicrobial peptides.

Name	Amino Acid Sequence ^a^	Origin	Reference
α-defensin (HNP-2)	C_1_YC_2_RIPAC_3_IAGERRYGTC_2_IYQGRLWAFC_3_C_1_	Human	[44]
β-defensin (BD2)	GIGDPVTC_1_LKSGAIC_2_HPVFC_3_PRRYKQIGTC_2_GLPGTKC_1_C_3_KKP	Human	[20]
LL-37	LLGDFFRKSKEKIGKEFKIVQRIKDFLRNLVPRTES	Human	[45]
Protegrin	RGGRLC_1_YC_2_RRRFC_2_VC_1_VGR	Pig	[46,47]
Indolicidin	ILPWKWPWWPWRR-NH_2_	Cattle	[34,35]
Magainin 2	GIGKFLHSAKKFGKAFVGEIMNS	African clawed frog	[48]
Cecropine A	KWKLFKKIEKVGQNIRDGIIKAGPAVAVVGQATQIAK-NH_2_	*Hyalophora cecropia*	[49]
Mellitin	GIGAVLKVLTTGLPALISWIKRKRQQ	Honey bee	[50]
Magainin II	GIGKFLHSAKKFGKAFVGEIMNS	African clawed frog	[10,11]
Polyphemusin	RRWC_1_FRVC_2_YRGFC_2_YRKC_1_R	Horseshoe crab	[5,51]
Gramicidin S	cyclo-(Val-Orn-Leu-D-Phe-Pro)_2_	*Bacillus brevis*	[52]
Nisin A ^b^	I-DHB-A_1_I-DHA-LA_1_-ABA_2_-PGA_2_K-ABA_3_-GALMGA_3_NMK-ABA_4_-A-ABA_5_-A_4_HA_5_SIHV-DHA-K	*Lactococcus lactis*	[53]

^a^ connected cysteines forming disulfide bridges are indicated with numbers in subscript; ^b^ nisin contains five lanthionine/β-methyllanthinonine rings between alanine and/or aminobutyric acid (ABA) residues, it also contains several dehydroalanine (DHA), dehyrdobutyrine (DHB) residues. The ring pattern is indicated with numbers in subscript.

Human cathelicidin LL-37 is an extensively studied member of the cathelicidin family of antimicrobial peptides, and is also the only cathelicidin found in humans. It is most abundant in the granules of neutrophils (cells of the immune system). Upon infection and inflammation, LL-37 is released in high concentrations at neutrophil accumulation sites. Human cathelicidin LL-37 is also produced by lymphocytes and macrophages as well as different epithelial cells, and hence detected in plasma, sweat and other body fluids [54]. The family of cathelicidins are classified based on their highly conserved cathelicidin domain, which is flanked by an *N-*terminal signal peptide and a *C*-terminal segment corresponding to the active antimicrobial peptide. Thus, LL-37 is the predominant fragment from proteolytic cleavage of the *C*-terminal of the human host defense precursor protein (hCAP-18) (Figure 1 and Table 1) [45]. The gene encoding hCAP-18 contains three vitamin D receptor elements in its promoter region and is under regulation by various signaling pathways where multiple receptors are involved [55,56]. Ligand binding to the vitamin D receptor, triggers complexation with vitamin D receptor elements in the promoter region, initiating transcription of mRNA that translates into hCAP-18 precursor protein [57,58]. LL-37 is up-regulated under inflammatory conditions and is also specifically up-regulated in response to compounds like butyrate and vitamin D_3_ [59]. Bacterial components are another type of LL-37/hCAP-18 gene expression inducers. For example, it has been shown that expression of LL-37/hCAP-18 significantly increased in *Helicobacter pylori-*infected patients in contrast to individuals with non-*Helicobacter pylori* induced inflammation [60]. Once expressed, LL-37 exerts various biological activities;* i.e.*, direct bactericidal activity against Gram-positive and Gram-negative bacteria observed *in vitro* [61] and modulation of inflammation and immune response in host cells against various infections [62,63]. LL-37 promotes apoptosis of infected epithelial cells, promoting clearance of respiratory pathogens [64]. Production of cytokines like interleukin-6 and interleukin-10 is also stimulated by LL-37 in a synergistic manner, thus enhancing the immune response through a complex mechanism involving multiple pathways [65]. Other studies have also shown that LL-37 enhances production of interleukin-8 and induces the expression of α-defensins, another class of cationic host defense peptides [66]. Enhanced clearance of *Pseudomonas aeruginosa*, an opportunistic pathogen, of immuno-compromised individuals has been demonstrated by LL-37 expression in murine lungs [67]. Besides stimulating production of immune signalling molecules, LL-37 can also function as a chemo-attractant, recruiting mast cells involved in wound healing and defence against pathogens, thus promoting the release of pro-inflammatory mediators [68]. In parallel to up-regulating several cytokines LL-37 can also inhibit interferon-gamma expression, a crucial cytokine for the innate and adaptive immunity against viral and bacterial infections [69]. Even though the fundamental mechanisms of LL-37 contribution to pathogen elimination have been extensively reported, the specific mechanisms by which LL-37 modulates the innate immune response remain poorly characterized.

## 3. Insect Antimicrobial Peptides

Insects represent by far the most diverse group of animals on Earth. Lacking an adaptive immune system, they fight against various pathogens by quick cellular and humoral responses. Thus, antimicrobial peptides play an important role in the insect’s defense system as they are secreted in the hemolymph as a result of the humoral defense mechanism upon microbial infections. Antimicrobial peptides isolated from different insect species are categorized as *i.e.*, cecropins, attacins, lysozymes, defensins and dipteracins. Furthermore, in *Drosophila* two new groups, drosomycin and the metchikowins, have been identified and characterized [70].

### 3.1. Cecropins

Cecropins are the most abundant class of linear antimicrobial peptides in insects. They are devoid of cysteine residues and adopt most often an α-helical conformation in membrane mimetic environments. To exemplify, cecropin A (KWKLFKKIEKVGQNIRDGIIKAGPAVAVVGQATQIAK-NH_2_) is one of the first cecropins for which a solution confirmation for adaptation of a stable α-helix in hydrophobic environments has been demonstrated using circular dichroism spectroscopy (Table 1) [49]. In general, the structural fingerprint of cecropins is the presence of tryptophan residues at position 1 and 2 in addition to the amidated *C*-terminus [71]. However, a few exceptional structures including non-amidated *C*-terminus and peptides without *N-*terminal tryptophans have also been reported, with broad spectrum antibacterial activity comparable to that of cecropin A [72].

### 3.2. Melittin

Melittin is the most characterized α-helical, cationic and hemolytic antimicrobial peptide in the literature, which constitutes 50% of the dry weight of bee venom [73]. It is a 26-amino acids amphiphilic peptide (GIGAVLKVLTTGLPALISWIKRKRQQ), characterized by a hydrophilic *C*-terminal and predominantly hydrophobic *N-*terminal region, and serves as a model peptide in many membrane-peptide interaction studies (Table 1) [50]. Moreover, structure-activity relationship studies where different segments of melittin have been exposed to substitution or deletion, have demonstrated the key elements required for the high hemolytic and antimicrobial activity of this peptide. For example, replacement of Pro_14_ with Ala yields a 2-fold higher hemolytic peptide [50], deletion of the 6 residues from the *C*-terminal end results in a non-hemolytic peptide fragment [74]. Also, deletion of specific residues such as Leu_6_, Leu_9_, Leu_16_, Iso_17_ and Trp_19_ causes decrease in the hemolytic activity and deletion of Ala_4_ and Lys_7_ reduces the antibacterial activity relative to the native melittin [75,76]. In regards to the mechanism of action, melittin is established as a pore forming antimicrobial peptide, though there is no universal agreement of the type of pore it forms (for a review see [73]). In an attempt to design small analogs of naturally occurring antimicrobial peptides, researchers have designed hybrid peptides consisting of elements from cecropin A and melittin, which have demonstrated very potent antibacterial activity and no hemolytic activities [77]. One of the hybrid peptides, cecropin A-melittin (CAM), has been further modified by replacement of four specific residues with Trp_5,11,21,25_ in order to improve the antibacterial activity and improve the proteolytic stability. Thus, the new hybrid, CAM-W exhibits 3–12 times higher antibacterial activity, and strong antifungal activity while retaining a moderate cytotoxicity (IC_50_ > 300 mg/L) [78]. In summary, native or modified insect antimicrobial peptides hold potential as alternatives to the conventional antibiotics against various bacterial and fungal pathogens.

## 4. Plant Antimicrobial Peptides

Plants have also evolved a plethora of defense molecules to fight pathogenic infections. Antimicrobial peptides isolated from plants are positively charged and display the typical structural motifs such as rtα-helices, β-sheets and loops. They are divided into five classes namely; lipid transfer proteins [79], thionins, defensins [80], chitin-binding proteins [81] and cyclotides [82]. Thionins and defensins represent the first antimicrobial peptides isolated from plants, thus their activities will be discussed in the following section.

### Thionins and Defensins

**Figure 3:**
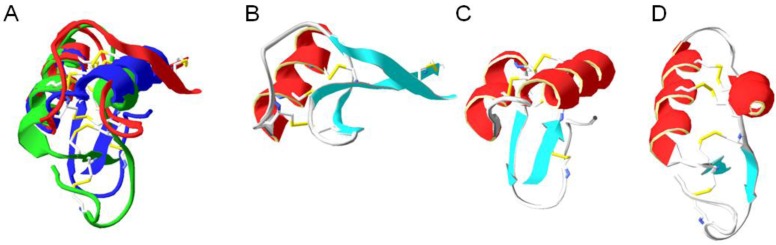
Three structural classes of thionins. (**A**) Members from all three classes have been superimposed to show their similarities. The same structures are illustrated separately in panels B-D. The PDB codes are all indicated, in addition to the primary sequence with the disulfide bridges indicated with subscript numbers; (**B**) β-purothionin (PDB code 1BHP) KSC_1_C_2_KSTLGRNC_3_YNLC_4_RARGAQKLC_4_ANVC_3_RC_2_KLTSGLSC_1_PKDFPK [87]; (**C**) γ 1-H thinonin (PDB code 1GPT) RIC_1_RRRSAGFKGPC_2_VSNKNC_3_AQVC_4_MQEGWGGG NC_2_DGPLRRC_3_KC_4_MRRC_1_ [88]; and (**D**) hellethionins (PDB code 1NBL) KSC_1_C_2_RNTLARNC_3_YNAC_4_RFTGGSQPTC_4_GILC_3_DC_2_IHVTTTTC_1_PSSHPS [89].

Thionins consist of up to 48 amino acids, predominantly arginine, lysine and cystein residues and contain a few conserved disulfide linkages (Figure 3). As a part of the plant defense system, thionins act on various pathogens and exert toxic effects on bacteria, fungi, yeast and mammalian cells [83]. A subgroup of the thionins family is the β-purothionins, which interact strongly with lipid bilayer, then insert into the hydrophobic core, resulting in cell lysis [84]. Viscotoxins, another class of plant thionins, were isolated from the leaves and stems of the European mistletoe (*Viscum album*) in 1973 [85]. Studies have demonstrated their high conformational stability and their ability to disrupt bacterial membranes [86]. γ-Thionins, now known as plant defensins, are another superfamily of plant antimicrobial peptides.

## 5. Antimicrobial Peptides Produced by Bacteria

Antimicrobial peptides produced by bacteria, originally named colicins, are referred to nowadays as bacteriocins. Bacteriocins produced by the host bacteria are able to selectively act against a broad spectrum of bacterial species without harming the producer. This family of antimicrobial peptides finds expansive application in the food industry to protect and prevent food contamination, particularly as many bacteriocins are produced by food-grade lactic acid bacteria. Additionally, bacteriocins attract special attention for their clinical implications for treatments of methicillin-resistant *S. aureus*, enterococci (including VRE), streptococci, *Clostridium botulinum* and *Propionibacterium acnes*. As a result of extensive work over the past decades there have recently been calls for the classification of bacteriocins to be revised and the current grouping suggested by Cotter *et al.* includes Class I (lanthionine-containing), Class II (non-lanthionine containing) and bacteriolysins (non-bacteriocin lytic proteins) [90].

### 5.1. Nisin

**Figure 4:**
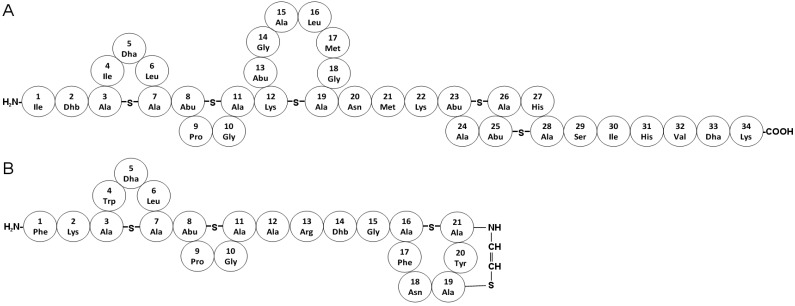
Covalent structures of two highly related bacteriosins. (**A**) Nisin A from *Lactoccocus lactis* and (**B**) Mutacin 1140 from *Streptococcus mutans*.

Nisin is a classical example of a Class I bacteriocin that has been successfully approved as an antimicrobial with commercial relevance in over 50 countries worldwide since its discovery back in 1927 [91]. Isolated from *Lactoccocus lactis* it was not until 1971 that its complex structure, consisting of 34 amino acids including some unusual lanthionine residues, four β-methyllanthionines, didehydroalanine and didehydroaminobutyric acid was fully described (Figure 4 and Table 1) [53]. Its activity against Gram-positive pathogenic bacteria is attributed to its interaction with the bacterial membrane resulting in disruption of the membrane integrity. This occurs via pore formation and so far two mechanisms are proposed. The first one explains the low-affinity pore formation of nisin alone, whereas the second one accounts of nisin and lipid II interaction thus ascribing to the pore formation by this complex (Lipid II-dependent pathway) [92,93,94,95]. Several structure-activity relationship (SAR) studies have been reported where the importance of different segments, especially those containing lanthionine rings, have been highlighted to be essential for the observed antimicrobial activity [96].

### 5.2. Mutacin 1140

Mutacin 1140 [97] is another member of the lanthionine-containing bacteriocins which has been extensively studied. It is a 22 amino acid long peptide produced by *Streptococcus mutans* [98] with a mode of action similar to that of nisin, which relates to the ability to inhibit peptidoglycan synthesis by binding to lipid II (Figure 4) [95]. Mutacin 1440 has a broad spectrum of activity against clinically important bacteria with time of kill profiles consistent with that of vancomycin, an antibiotic approved for treatment of severe infections caused by Gram-positive bacteria. This peptide is currently under investigation for its pharmacological relevance and has entered preclinical studies [99].

Lysostaphin is a bacteriolysin that acts by hydrolyzing the cell wall of the pathogenic bacteria, thus it is currently being investigated for its potential in food and nutritional microbiology. Even though bacteriolysins are structurally much larger than the traditional bacteriocins and antimicrobial peptides, their application as a peptide antibiotics hold vast potential. Lysostaphin (27 kDa) for example, is able to cleave the pentaglycine cross-links in the cell wall of *Staphylococci* and thus lyse the pathogenic bacteria in all metabolic states. The therapeutic efficacy of this bacteriolysin against clinical methicillin-resistant *S. aureus* MRSA isolate has been reported in animal models and compared to that of vancomycin. The recombinant lysostaphin, displayed better *in vitro* and *in vivo* antibacterial activity against methicillin-resistant *S. aureus* compared to vancomycin, in addition to having a low toxicity profile, therefore it is a potential lead candidate for further structural optimization studies [100].

## 6. Structural Properties of Antimicrobial Peptides

The structural properties of any given biomolecule play a major role in the interpretation of its biological activity. Many studies have been conducted to dissect the important elements that define peptides as antimicrobial “weapons”. Broad diversity of antimicrobial peptide sequences exists in nature, which contributes to the overall structural diversity, yet there are evolutionarily traits that have been conserved to ensure their activity on various types of bacteria with different membrane composition and different targets. The secondary structure, cationicity, hydrophobicity and amphipathicity are the most important key elements that allow characterization of antimicrobial peptides and therefore are briefly discussed in the following section.

### 6.1. Secondary Structure

Based on their secondary structures, antimicrobial peptides are classified in four groups; α-helices β-sheets, mixed structures and non- α- or β- structures (extended) (Figure 1). Circular dichroism spectroscopy, X-ray crystallography and nuclear magnetic resonance spectroscopy have been used intensively for the structure determination of antimicrobial peptides. For example, the first X-ray crystallographic structure of native human α-defensin, the human neutrophil peptide 3, appeared in 1991, followed by a nuclear magnetic resonance structure of human neutrophil peptide 1 [101,102,103]. Such structural information is found to be useful when investigating the importance of the secondary structure in deciphering the antimicrobial activity of antimicrobial peptides. To exemplify this, studies have demonstrated that bacterial killing by human neutrophil peptide 1 is structure-independent [104]. The most common motif found in many proteins and peptides with biological activities is the amphipathic α-helix, however, many antimicrobial peptides exist as extended or unstructured conformers and only adapt α-helical conformations upon interaction with phospholipid membranes [105,106]. Circular dichroism analysis has shown that in the presence of unilamellar phospholipid vesicles with varied content of zwitterionic and negatively charged phospholipids, several antimicrobial peptides adopt well defined α-helical and/or β-sheet like structures in contrast to the buffer environment [107].

The presence of α-helical structures in antimicrobial peptides is generally believed to promote interaction with membranes and assist membrane lysis [108] and therefore specific amino acids such as alanine, leucine, arginine, lysine, *etc.*, that have high helical propensity (they occur more frequently in α-helices) have been actively included in the design of potential novel antimicrobial peptides [109,110]. In this context, Deslouches *et al.* showed that in general, the peptides from their de novo library, with more than 80% helical content, exhibited maximal antimicrobial potency against the tested bacterial strains [111]. On the other hand, Javadpour *et al.* also designed a library of highly α-helical peptides containing lysine and/or leucine/alanine/glycine residues in sequences that extended up to 21 residues. In this library the propensities of α-helical conformation was found to be proportional to the toxicity of the peptides against mammalian model cells. However, no definite conclusion could be obtained for a correlation of these peptides’ conformations and their observed antibacterial activity [112].

### 6.2. Conserved Salt Bridges

**Figure 5:**
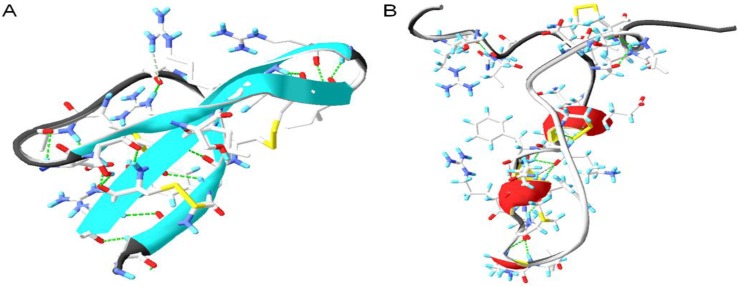
Salt bridges in antimicrobial peptides. The overall secondary structure is depicted, in addition to side-chains and back-bone elements that are involved in stabilizing the structure; hydroben bonding (green dotted lines) or salt bridges (grey dotted lines). The PDB codes are all indicated, in addition to the primary sequence with the disulfide bridges indicated with numbers in subscript. (**A**) On human defensin 5 (PDB code 2LXZ) ATC_1_YC_2_RTGRC_3_ATRESLSGVC_2_EISGRLYRLC_3_C_1_R [115], it is apparent how Arg_6_ and Arg_28_ from β-strand 1 and 3, clam around and stabilize the random coil backbone through formation of one hydrogen bond and one salt bridge; (**B**) Human lactoferricin 1-49 (PDB code 1Z6V) GRRRRSVQWC_1_AVSQPEATKC_2_FQWQRNMRKVRGPPVSC_2_IKRDSPIQC_1_IQA [114], contains a very loose structure with a disrupted or segmented helical elements. The composition of the helical element is pending on how the structure is captured, and as depicted in this structure, it is apparent how a salt bridge between Lys_19_ and Gln_24_ are involved in stabilizing the random coil bridging the two largest helical segments.

Salt bridges can be found in both α-helical and β-sheet peptides, and contribute greatly to the overall stability of the secondary structure of antimicrobial peptides. However, studies on α-defensin has demonstrated that salt bridges in human defensin 5 do not contribute to the antimicrobial activity, but rather affect the correct disulfide pairing of the pro-α-defensins, which are inactive defensins subjected to further processing. Furthermore, the salt bridges can also indirectly increase proteolytic stability, as seen for human defensin 5 (Figure 5), where modifications that disrupted the salt bridges also accelerated degradation by trypsin [113]. Similar salt bridges has also been observed in other antimicrobial peptide structures, e.g., in human lactoferricin 1-49 (Figure 5) [114].

### 6.3. Cationicity

One common property of the majority of the antimicrobial peptides is the cationic nature represented by the number of positively charged residues (lysine, arginine and histidine) within their structure, which ranges between +1 and +7 charges [3]. A number of studies have correlated this property with the antimicrobial activity observed in antimicrobial peptides. The attributed importance lies mainly in the interaction between the positive charge in the peptides and the negatively charged bacterial membrane surfaces via electrostatic interactions. For instance, the antimicrobial activity of most of the members of the defensin family appears to be related to their cationicity. To exemplify, human defensin 5 interacts with the bacterial surface via its arginine residues and thus exerts its antimicrobial activity. Replacement of arginine residues at position 9 and 28 with alanine or lysine residues reduces the antibacterial killing as well as the host cell interaction, the latter which is found to be receptor mediated [116]. The overall positive charge may not be the main determinant in the observed antibacterial activity as in case of human α-defensins that generally show to be more effective against Gram-positive bacteria than human β-defensins, even though human β-defensins are more cationic [117,118]. Herein, human neutrophil peptide-1 (ACYCRIPACIAGERRYGTCIYQGRL WAFCC, net charge +3) appeared to be more effective against *S. aureus* than human β-defensin-3 (GIINTLQKYYCRVRGGRCAVLSCLPKEEQIGKCSTRGRKCCRRKK net charge +11) [23,119,120]. In addition, cationicity meets a limit, beyond which increasing the charge no longer results in increased antibacterial activity. That being said, Dathe *et al.* has demonstrated that increasing the charge from +3 to +5 in magainin-2 analogues, also increased the peptides antibacterial activity against both Gram-positive and Gram-negative bacteria, but further increase to +6 or +7 led to a loss of the antibacterial activity and increased hemolytic propensity [121]. In addition to the net charge of antimicrobial peptides, it has further been illustrated that the position of the charged residues is also an important factor that determines the peptides overall antibacterial activity, e.g., changing the position of a few amino acid residues within the native structure of the linear bactenecin, Bac2A (RLARIVVIRVAR-NH_2_), resulted in a scrambled sequence that showed increased antibacterial activity [122]. Besides the importance of cationicity in mediating initial interaction with target membranes, in Gram-negative bacteria, the net positive charge is important for the so-called self-promoted uptake of antimicrobial peptides. Herein cationic antimicrobial peptides interact with the outer membrane surface where there are divalent cations such as Mg^2+^ or Ca^2+^ cross bridging LPS molecules. Displacement of these cations causes destabilization of the outer membrane and therefore allowing uptake of molecules [123,124]. Moreover, bacteria carry more negative transmembrane potential when compared with that of a normal mammalian cell membrane and this will facilitate insertion of charged antimicrobial peptides into the membranes [125].

### 6.4. Hydrophobicity

Hydrophobicity is certainly an inevitable structural feature that decides the overall activity of a given antimicrobial peptide and therefore it is continuously characterized in the literature as a key functional property. It affects the potential of interaction between antimicrobial peptides and different membrane compositions and furthermore directs the degree of peptide partitioning into the lipid bilayer. Increased hydrophobicity is well correlated with loss of antibacterial specificity, resulting in high toxicity towards mammalian cells. To elaborate, Yin *et al.* showed that substituting four alanine residues with four hydrophobic leucine residues induced higher hemolytic activity of their membrane active model peptide [126]. Magainin-2 analogues with varying hydrophobicity have also been used to demonstrate that minor changes in the hydrophobicity may drastically influence and increase its membrane binding and permeabilization activity [127]. The effect of hydrophobicity on the antibacterial and hemolytic activities has been further demonstrated via peptide series constructed of repeats of lysine and tryptophan (LysTrp)_n_ residues. Addition of a number of repeats of these two residues resulted in parallel increase of both hydrophobicity, as estimated by the retention times on HPLC, and antimicrobial activity. However, when five repeated units were present in the structure (LysTrp)_5_, the increase caused unfavorable change in the hemolytic profile (increased toxicity) and decrease in the antimicrobial activity due to self-aggregation [128]. In conclusion, the hydrophobicity of a specific sequence in the development of a novel antimicrobial peptide should be challenged but not exaggerated.

### 6.5. Amphipathicity

Amphipathicity in antimicrobial peptide structures reflect the abundance and polarization of the hydrophobic and hydrophilic domains. Most of the cationic antimicrobial peptides display a net positive charge (+1 to +7) [3] and consist of about 50% hydrophobic residues which contribute to the recognition and interference with the cytoplasmic membrane barrier or self-promoting uptake across the cellular membranes. The cationic charges and the hydrophobic groups segregate into amphiphilic structures. One quantitative measure of amphipathicity is the hydrophobic moment, that applies for peptides in α-helical confirmation and is used as a descriptor in dissecting the role of amphipathicity for peptide antimicrobial activity [129]. The importance of the amphiphilicity in determination of the antimicrobial activities of these peptides is controversial because different research groups report on favorable and unfavorable contributions such as increase in antimicrobial and increase in hemolytic activities, respectively [127,130,131,132,133,134,135]. For example, amphipathicity has been reported as a major structural determinant for the biological activity of a small library of arginine and tryptophan rich linear and cyclic hexapeptides [133]. Here, the structural and conformational constrains of the peptides together with the ideal positioning of the hydrophobic clusters appeared to determine the antimicrobial activity and selectivity of the peptides [133]. In another study of magainin-2 and its analogues it was demonstrated that the antimicrobial activity was governed explicitly by the peptide amphipathicity and not by hydrophobicity or α-helical content [134]. Similarly, a design of cecropin A and melittin hybrid structures has demonstrated increased amphiphilicity and helicity correlating with high antibacterial activity and low toxicity against mammalian cells [135]. Contradicting these results are numerous other peptide studies, demonstrating that high amphipathicity if measured as hydrophobic moment, increases membrane disruption resulting in increase in both the antibacterial and hemolytic activity [127,130,131]. Amphipathicity in antimicrobial peptides that exist in β-sheet conformations is characterized by the number of β-strands organized by two distinct polar and non-polar domains. β-strands in antimicrobial peptides are usually stabilized via disulfide bridges or head-to-tail cyclization which provides high conformational rigidity in aqueous solution. The polar and non-polar domains in β-strands allow antimicrobial peptides to successfully interact with target membranes and once associated with the membrane, the amphipathic nature enables membrane disruption via formation of transmembrane channels [105]. Alteration in the non-polar domain of gramicidin S derivatives, decreased hydrophobicity and the overall amphipathicity, thus not surprisingly decreased the peptides hemolytic activity [136].

### 6.6. Cyclic Antimicrobial Peptides

**Figure 6:**
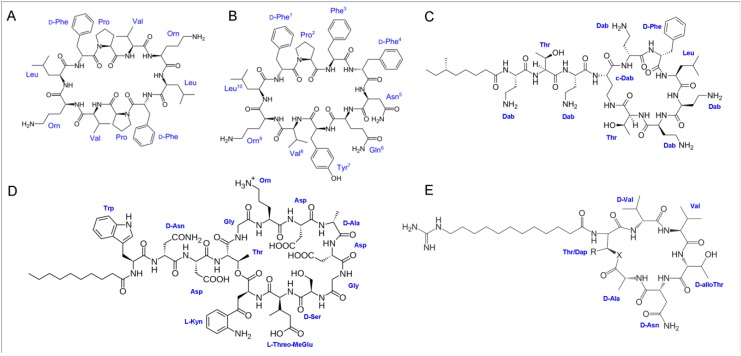
Cyclic peptides and lipopeptides. Covalent structures of some conventional and lead drug molecules. (**A**) Gramicidin S; (**B**) Tyrocidines; (**C**) Polymyxin B; (**D**) Daptomycin and (**E**) cyclo-[D-Ala-(12-guanidinododecanoyl)Thr-D-Val-Val-DaThr-D-Asn] [145].

Besides adopting linear structures, antimicrobial peptides can be naturally found in cyclic conformations. Antimicrobial peptides are constrained in this conformation either by disulfide cross linkages (tachyplesins (KWC_1_FRVC_2_YRGIC_2_YRRC_1_R-NH_2_) [137], protegrins (RGGRLC_1_YC_2_RRRFC_2_VC_1_VGR-NH_2_) [138], and polyphemeusins (Figure 1)) or backbone cyclization (gramicidin S, tyrocidines (Figure 6) and θ-defensins (Figure 2)). Cyclic antimicrobial peptides have demonstrated strong antimicrobial activities against different pathogenic bacteria, however with poor selectivity. Therefore, numerous structure-activity relationship studies have been done for dissecting the important elements that contribute to the observed activities with the intention to improve their therapeutic profiles [136]. Scheinpflug *et al.* demonstrated improved antimicrobial activity against Gram-negative and Gram-positive bacteria and low hemolytic activity of a small cyclic hexapeptides (c-RRRWFW), when compared to that of the linear analog sequence. Regarding the mechanism of action, this cyclic peptide did not act on the bacterial membrane, rather it has been suggested that it travers the cell wall with the help of direct interaction to lipopolysaccharide [139] and then further translocates to the cytoplasm [140]. Other studies have used the native structure of the gramicidin S, as a model peptide for synthesis of novel cyclic antimicrobial peptides [141]. In this context, the effect of the ring size (4-14 residues) in gramicidin S on the antimicrobial and hemolytic activity had been analyzed, in addition to the size effect on the secondary structure and the peptides lipid binding potential. The ring structures containing 6, 10 and 14 residues folded in β-sheet structures, whereas disordered structures had been observed for rings formed by 8 or 12 residues. The membrane disruption had been observed only in the peptide analogues with 10 or more residues in the ring structure, whereas those with less appeared to be completely inactive without any hemolytic activities. Out of this study only one cyclic analog showed improved antibacterial specificity, indicating that varying native cyclic peptide structures can contribute to the development of clinically useful cyclic peptide antibiotics [142]. The 14-mer analogue, GS14 forms a highly amphipathic β-sheet structure and has served as a model peptide for further modifications and structure activity studies. GS14 exhibits very high hemolytic and limited antimicrobial activity so any change in the structure that will disturb the amphiphilic β-sheet structure gives a direct relation of the amphipathicity and the biological properties of the cyclic peptide. By systematic synthesis of GS14 variants, Kondejewski *et al.* demonstrated that the hemolytic activity exhibited by GS14 could be easily reduced by decreasing the amphipathicity (reduction of directed hydrophobicity) or by reduction of the overall peptide hydrophobicity [136]. Other attempts to identify structural features and improved antimicrobial activity of cyclic peptides have been communicated in two consecutive studies [133,143]. Here, the cyclic peptides with the highest antimicrobial activity were those with three aromatic residues positioned adjacent to each other. In summary, since amphipathicity is of a great importance when tuning the antimicrobial activities of peptides, cyclic peptides permit induced amphipathicity and greater enzymatic stability [43,144] and should therefore be considered a valuable path forward.

### 6.7. Length

The length of the polypeptide chain in antimicrobial peptides varies greatly and there are continuous reports on many active sequences that are not necessarily similar in size. This raises the question whether or not there is an optimal chain length that is beneficial for the observed activity of antimicrobial peptides. To answer this question, a few research studies are presented. For example, in design of a *de novo* peptide library it was demonstrated that by increasing the chain length of a 12-mer peptide composed of only arginine and valine, one could achieve an increase in antimicrobial activity against *P. aeruginosa* and *S. aureus* [111]. Notably, others have reported that an increase in length could give a proportional increase in the toxicity towards mammalian cells [112]. Similarly, Lui *et al.* showed that by increasing the chain length of a peptide from a 2-mer (RW) to a 10-mer (RW)_5_ both the antibacterial activity and host cell toxicity increased, however the latter being less affected [146]. Correspondingly, in a study with α-helical model peptides (lysine, leucine, alanine), it appears that 21-mers in general are about two-fold more potent than their 14-mer analogues. Contradictory observations have been made in a different α-helical model peptide library (LARL)_3_-(LRAL)_n_ (*n* = 0–3). In this study the peptides exhibited decreased antimicrobial activity and yet increased hemolytic activity with increasing chain length [147]. The above observations are further supported by a study by Gopal *et al.* where an increase in chain length up to eight residues enhanced antimicrobial activity of peptides composed of repeated lysine and tryptophan (KW)_n_ and the effect on hemolysis increased gradually with the increase of the chain length. However, the increase of chain length from (KW)_4_ to (KW)_5_ did not further improve the antimicrobial activity [128]. A similar trend demonstrating increase in antibacterial activity up to a certain chain length before it decreases again, has also been reported for a set of arginine and valine rich β-hairpin like peptides (Ac-C(VR)_n_^D^PG(RV)_n_C-NH_2_ (*n* = 1–5) [148]. Additional insight into peptide length can also be learned from peptide sequence truncation. Some examples include truncation of peptide LL-37 [149]. Thus, in conclusion there is no definite length of a peptide sequence that is to be taken into account when one designs novel antimicrobial peptides. However, the studies conducted provide a better understanding of this issue and suggest optimal strand length to improve antibacterial peptide activity for the respective peptide species studied.

## 7. Structural Properties of Specific Amino Acid Residues in Antimicrobial Peptide Sequences

Identified antimicrobial peptides from plants, insects, fish, frogs and mammals vary greatly with respect to their amino acid composition. Each of the amino acids alone or in synergy with the neighboring amino acid residues contributes to the observed antimicrobial properties conserved in the peptides. The following section highlights the importance of few amino acids commonly found in the structure of many cationic antimicrobial peptides and discusses their contribution to the peptides therapeutic profiles.

### 7.1. Lysine (Lys, K) and Arginine (Arg, R)

The basic amino acids Lys and Arg are highly conserved residues in antimicrobial peptide structures as they enable electrostatic interactions between the peptide and the negatively charged bacterial membranes [150]. These two amino acids differ in their side chain chemistry. Arginine has a guanidinium group that allows more dispersed positive charge and offers greater directionality and possibility of hydrogen bonding with for example the surrounding water molecules. The unique characteristics of the arginine side chain allow formation of multiple interactions contrasting the mono charge present in lysine. In addition, arginine can also engage in cation–π interaction with tryptophan, where the negative charge clouds in the tryptophan aromatic systems interact with the positively charged side chain [151]. The roles of amino acid substitution and cationicity on antimicrobial and hemolytic activities have been thoroughly investigated over the past 20 years. For this reason, many peptide analogues have been synthesized where known sequences have been modified to include different cationicity using lysine and arginine. Many studies have demonstrated that substitution of arginine with lysine leads to reduced antimicrobial activity [152,153]. Gopal *et al.* [154] reported on an analogue sequence, (FKKLKKLFKKILKLK-NH_2_) of HPA3NT3 peptide, where they substituted Trp_12_ and Trp_14_ with leucine as well as Arg_3_ and Asn_13_ with lysine. They observed an increase in the cationicity due to the substitution of arginine and asparagine with lysine and *C*-terminal amidation, which resulted in small changes in the antibacterial activity but significant decrease in hemolytic activity [154]. Similarly, substitution of arginine with lysine in cyclic c-RRWWRY resulted in decreased minimum inhibitory concentration and erythrocyte lysis [143]. In addition to the contribution of arginine residues to the initial electrostatic recognition of the membrane surfaces, poly-arginine sequences are able to pass cell membranes more efficiently than poly-lysine or other poly-cationic homopolymers, suggesting that the guanidine group of the arginine side chain is a critical component for the observed biological activity. Furthermore, once the peptides have entered the cell, arginine containing peptide sequences have demonstrated higher affinity for DNA than poly-lysine peptides [155]. Similarly, the arginine-rich peptide (RRWWRRWRR) has been actively used in the intracellular delivery of peptide nucleic acids. Substituting arginine residues in this sequence with lysine decreased the cellular uptake of the conjugate by six-fold [156]. Arginine- and lysine-rich peptide sequences are well described in histones, proteins found in eukaryotic nuclei that contribute to package and organization of DNA into nucleosomes. Lysine and arginine rich histones and histone fragments exhibit broad-spectrum antimicrobial activity [157] as a result of intracellular inhibition of cell functions trough binding of these fragments to bacterial nucleic acids [158,159]. Consequently, Morita *et al.* demonstrate that the contribution from these fragments to the observed antibacterial activity relies on the arginine-rich histone ability to disrupt the morphology and lysine-rich histone to disrupt the integrity of *S. aureus* and *E. coli* [157,159]. One of the limitations of the extracellular applications of antimicrobial peptides as antimicrobial coating agents is the inhibition of the electrostatic interactions with increasing concentrations of sodium chloride. To counteract this limitation, incorporation of arginine residues at the *C*-terminal end of antimicrobial peptides allows salt-resistance due to the increased cationicity which overall will improve the electrostatic interaction between the peptide and the anionic bacterial membranes [160,161]. To exemplify, human β-defensin has been exposed to such structural changes that led to improved antibacterial potency in ionic environments and further boost the development of many antimicrobial peptides that deal with such obstacles [160]. While tuning the membrane selectivity of antimicrobial peptides, Liu *et al.* [162] reported on another arginine-rich peptide sequence, (RW)_4D,_ with enhanced antibacterial activity against *E. coli* and *S. aureus* and lowered hemolytic profile when compared with the natural antimicrobial peptide indolicidin [162]. Another example of improved membrane permeability of arginine containing peptide sequences is the simple substitution of D*-*lysine with D-arginine in the short antimicrobial peptide RLA [163].

### 7.2. Tryptophan (Trp, W)

Another very important residue found in the structure of antimicrobial peptides is tryptophan. The unique side chain containing an indole ring holds hydrogen-bonding potential in addition to other physiochemical properties, e.g., dipole and quadrupole moments [164]. Tryptophan residues show strong membrane-disruptive activities by their ability to interact with the interface of a membrane anchoring the peptide to the surface of the bilayer [165]. Scanning electron microscopy analysis has suggested that two tryptophan-substituted antimicrobial peptides, I1WL5W and I4WL5W, work through disruption of the bacterial cell membranes. In addition, fluorescence and quenching data from liposome studies has indicated possible insertion of these peptides into the lipid bilayers and induction of blue shifts in the tryptophan emission spectra [166]. The hydrogen bonding of tryptophan with the surrounding water molecules diminish upon insertion of the tryptophan residues into the hydrocarbon core of the membrane [167]. However, indole hydrogen bonding and the dipole-dipole interactions are shown not to be primary determinants of the tryptophan interfacial localization [167]. Various experimental and molecular-dynamic simulation studies have demonstrated accommodation of tryptophan residues in the interface layer of membranes [167,168] which could associate with the positively charged choline head groups of the lipid bilayer [151]. Overall, it is suggested that aromaticity and the molecular shape play an important role in explaining the nature of membrane interaction of tryptophan-rich antimicrobial peptides. To exemplify, the flat and rigid structure of tryptophan may influence the positioning in the hydrocarbon core for entropic reasons and the electrical properties of the aromatic system may favor accommodation of tryptophan in the interface regions. In accordance with a plethora of literature studies pointing out the importance of the overall structure and position of certain amino acids in antimicrobial peptide design, the position of tryptophan residues in the tryptophan substituted peptide, *L-*K6, a peptide derived from temporin-1CEb from skin secretions of the Chinese brown frog, demonstrated to be an important factor for the observed antibacterial activities against Gram-negative and Gram-positive bacteria [169]. The influence of tryptophan residues on antimicrobial potency against *P. aeruginosa* and *S. aureus* has been further documented where single substitution with tryptophan significantly increased the anti-pseudomonal potency when compared to the parent peptide [111]. In summary, tryptophan residues have strong preference for the interfacial regions of the lipid bilayer and aid attachment and insertion of the tryptophan containing peptide across the membrane barriers.

### 7.3. Cysteine (Cys, C) and Disulfide Bonds

Cysteine belongs to the sulfur-containing amino acids which are strongly reactive. The thiol group can be easily oxidized to form a dimer, thus creating a disulfide bridge between two cysteines. Disulfide bridges formed by cysteine residues are strongly hydrophobic (nonpolar) and play an important role in the structures of many antimicrobial peptides. In addition to being important for the overall structural fold of the peptide, these bridges also increase the peptides stability towards proteolytic degradation, e.g., defensins [170]. The tertiary structure stabilization via disulfide bonding of human neutrophile peptide 1 (HNP1) contributes to effective binding to the cell wall precursor lipid II [104] and inhibits TNF-α secretion by human monocyte derived macrophages [171]. In studies where disulfide bonds in HNP1 or human defensin 5 (HD5) were reduced (e.g., by dithiothreitol) or substituted, it has been demonstrated reduced antibacterial activity [170,172]. In contrast, in the highly cationic (+11) human β-defensin 3 (HBD3), the presence, absence or altered pairing of the three disulfide bridges, did not appear to be relevant for the observed antibacterial activity [173]. Some of the most potent natural cysteine containing antimicrobial peptides, are a β-hairpin polyphemusin 1 (RRWC_1_FRVC_2_YRGFC_2_YRKC_1_R) from horseshoe crab (Figure 1) [5,51] and pig protegrin (RGGRLC_1_YC_2_RRRFC_2_VC_1_VGR) [46,47]. Polyphemusin I exhibit high antimicrobial activity with MIC ranging from 0.125 to 1 µg/mL against multiple clinical strains of both Gram-negative and Gram-positive bacteria [174]. Similarly, protegrins, especially protegrin-1 is also stabilized by two internal disulfide bridges (Cys_6_-Cys_15_ and Cys_8_-Cys_13_) and as such is able to permeabilize bacterial membranes including the outer membrane of Gram-negative bacteria [175,176,177]. Loss of bactericidal activity is observed upon removal of cystein residues, especially those in position Cys_6_ and Cys_15_ [178,179]. Structural studies have demonstrated that protegrin-1 has the ability to form oligomeric β-sheet like structures in model membranes, inducing pore formation in bacterial membranes [180,181,182]. Structure-activity relationship studies on protegrin-1 have been conducted to evaluate the importance of the cysteins, and the results demonstrated that two truncated linear versions of protegrin-1 retained a broad spectrum activity by disrupting LPS-outer membrane barrier. Furthermore it is assumed that the peptide adopts an amphipathic β-hairpin-like conformation, even in absence of the disulfide bonds in complex with the LPS micelles. Currently, protegrin-1 analogues are the main cysteine containing peptides that hold promise for development of a non-toxic antimicrobial [183]. One example is the protegrin-1 derivative IB-367 (iseganan) that has been the most studied for an effective treatment of oral mucositis due to its broad spectrum antibacterial activity, rapid killing and relative lack of resistance development. However this analogue failed in Phase II clinical trials of oral mucositis [174,184,185].

### 7.4. Proline (Pro, P)

Proline-rich antimicrobial peptides are a distinctive class of cationic peptides isolated from both insects and mammals, with confirmed antimicrobial activities specifically against Gram-negative bacteria [186,187,188]. Structurally, proline is an unusual amino acid that forms a ring structure with rigid confirmation and a secondary amine compared to the other twenty natural amino acids. This significantly reduces the structural flexibility of the polypeptide chain, and the nitrogen in the idole ring cannot participate in hydrogen-bonding with other residues. Prolines are often considered helix breakers, however, proline-rich sequences tend to adopt distinct PP-II helix (e.g., PR-39 (RRRPRPPYLPRPRPPPFFPPRLPPRIPPGFPPRFPPRFP), apidaecins, drosocin), helix structure with three residues per turn [189]. Retaining highly potent antimicrobial activities, proline-rich antimicrobial peptides subsequently act in a divergent way including stereospecific interaction with membrane translocation system followed by intracellular targeting, compared with the more general membrane disruption mode of action of traditional antimicrobial peptides. Most of the knowledge about the structure-activity relationship for these peptides comes from peptides isolated from insects; e.g., apidaecin, a small 18 amino acid residues peptide (GNNRPVYIPQPRPPHPRL) [190] isolated from honeybees, with proline content of 33% [191], drosocin, a 19-mer glycopeptide (GKPRPYSPRPTSHPRPIRV) [190] isolated from *Drosophila* [192] and pyrrhocorycin, a glycopeptide (VDKGSYLPRPTPPRPIYNRN) [193] isolated from the firebug *Pyrrhocoris apterus* [194,195]. In addition, Bac7, Bac5 and PR-39 are representatives of well-studied mammalian proline-rich antimicrobial peptides. Regarding the mechanism of action of these distinct peptides, studies have demonstrated that the members of the proline-rich peptide group and their derivatives act in a completely divergent mechanism than the lytic amphiphilic antimicrobial peptides [192,196,197,198,199]. To exemplify, one of the early observations of the non-lytic mechanism were found in *E. coli* challenged with apidaecin or bovine PR-39, where the bacteria membranes remained intact during the entire incubation time [188,191]. Similarly, arasin-1, a 37 amino acid long proline-rich peptide (SRWPSPGRPRPFPGRPKPIFRPRPC_1_NC_2_YAPPC_2_PC_1_DRW) [200] isolated from the spider crab, *Hyas araneus*, exhibits bactericidal antimicrobial activity not related to membrane disruption, with the proline-rich region (1–23) as the confirmed region responsible for the observed activity [201]. It has been further suggested that proline-rich antimicrobial peptides stereo specifically bind to intracellular targets such is the bacterial heat shock DnaK protein and this binding can be correlated with the observed antimicrobial activity [193,202]. Such observations were made for pyrrhocoricin, and the *N-*terminal half (Asp_2_-Pro_10_) were demonstrated to be responsible for the antimicrobial activity, while the *C*-terminal part aids internalization into bacterial or mammalian cells [193,195]. This proline-rich antimicrobial peptide protects mice from bacterial infections and is nontoxic to the host. Further studies on artificial proline-rich peptides has verified that a number of proline sequences can travers bacterial membranes, e.g., poly-proline (P_14_) peptide [203] as well as a polyprolines family of peptides (VXLPPP)_n_ (*n* = 1, 2, and 3; X correspond to histidine, arginine or lysine) [198]. Contrary to these findings there are also reports of proline-rich peptides and their ability to damage cell membranes, however these effects are highly concentration dependent [201,204,205].

Despite the well-established knowledge about the DnaK being an intracellular target for proline-rich peptides, very few studies suggest other vital intracellular targets in Gram-negative bacteria (for a review see [186,187]). Regardless, in the past five years, researchers have conducted new experiments to introduce proline-rich peptides to the market as promising antibiotics. Analogues of the native peptides, novel structures and synergistic studies between peptides and classical antimicrobials have been conducted [196,199]. In the efforts to design novel pyrrhocoricin derivatives peptides, large libraries have been synthesized and tested. One of the candidate peptides from this study, onconin (VDKPPYLPRPRPPRRIYNR-NH_2_), shares 70% structural similarity with pyrrhocoricin. It exhibited low toxicity towards mammalian cells and could penetrate lipid membranes without disrupting them [206]. Similarly Fritsche *et al.* [207] demonstrated that the antibacterial effect exerted by novel onconin and apidaecin derivatives, were not a result of immunomodulatory properties but rather a direct antimicrobial effect. An observation which potentially simplifies the development of novel proline-rich antimicrobial peptides into new pharmacological molecules [207]. Consequently and most recently, Krizsan *et al.* [208] speculate that onconins and apidaecins act on other targets than the chaperone DnaK alone [208]. In summary, proline-rich peptides are characterized by good water solubility, high potency against bacteria killing and low cytotoxic effects at high concentrations, making them attractive lead candidates for development of novel antimicrobial therapeutic agents.

## 8. Mechanism of Action of Antimicrobial Peptides

In drug development, a good antimicrobial candidate should exhibit highly specific biological activity followed by good pharmacokinetic profile and low immunogenicity. In order for peptides to be considered as antimicrobial agents of therapeutic relevance, it is essential to dissect their biological activities, specifically their mode of action. This task is not easy as there are over 2000 natural peptides with broad spectrum antimicrobial activities have been isolated. Despite this fact, a surplus of scientific study groups have provided us with structure-activity relationship information that helps the overall understanding of how antimicrobial peptide combat infectious agents. In order to do so, researchers have developed various model membranes that antimicrobial peptides act on. These include, micelles, artificial liposomes with varying lipid compositions (POPE, POPC, POPG, cholesterol *etc.*) and natural *E. coli* polar lipid extracts [209,210,211,212].

### 8.1. Direct Killing

**Figure 7:**
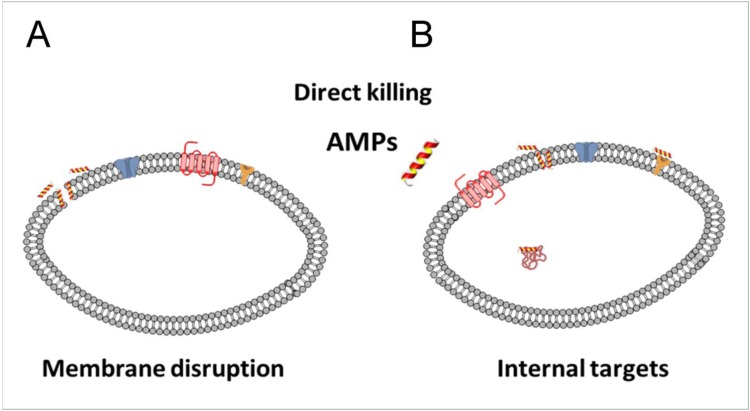
Direct killing mechanisms of antimicrobial peptides. The peptides are typically classified as membrane disrupting (**A**) or as molecules that act through more specific extra- or intracellular targets (**B**).

It is generally assumed that cationic antimicrobial peptides interact with membranes where they disturb the amphipathic lipid bilayer, which further leads to disruption of vital bacterial physiological processes and ultimately bacterial death. Bacterial membranes possess large fraction of negatively charged lipids and maintain high electrical potential gradients (transmembrane potential), thus attracting positively charged compounds such as cationic antimicrobial peptides. A result of the electrostatic interaction of positively charged peptides on the negatively charged bacterial surfaces, is destabilization and release of native divalent cations from the membrane. This displacement leads to disruption of the outer membrane barrier in Gram-negative bacteria (Figure 7).

In comparison to bacterial membranes, plant and animal cell membranes are enriched in cholesterol and lipids, have no net charge, and maintain weak transmembrane potential [213]. Studies that refer to the electrostatic interactions between the positively charged amino acids and the negatively charged phospholipid head groups in the membranes have shown that by increasing the buffer salt concentrations the peptide activity on the negatively charged membranes is diminished [214].

### 8.2. Membrane Interaction

Antimicrobial peptides share common physiochemical features such as cationicity and amphipathicity that allows them to interact with membranes. When peptides come in a close proximity to a model membrane (bacterial or mammalian), the very first interaction is the binding. Information about the strength of this interaction helps in understanding the consecutive processes that eventually lead to cell death.

Isothermal titration calorimetry is one of the techniques applied to extract information about the antimicrobial peptide binding affinity to model membranes. Andrushchenko *et al.* [212] investigated the thermodynamics of the interaction of tryptophan-rich antimicrobial peptides with large unilamellar vesicles (LUVs). They obtained binding isotherms that could characterize the strength of binding of the peptides to the model membranes. Specifically, all peptides demonstrated to selectively bind stronger to anionic (PE/PG) and *E. coli* membranes than to zwitterionic (POPC) membranes, due to strong electrostatic interactions. An increase in charge did not improve on binding and replacement of arginine with lysine residues did not change the binding to the anionic membranes but decreased the binding to the zwitterionic LUVs. Other substitutions like proline by alanine in one of the tryptophan-rich peptides allowed α-helix formation of the peptide that resulted in reduced binding selectivity. Furthermore, tryptophan substitution with other aromatic residues significantly reduced the peptides’ ability to interact with both the anionic and the zwitterionic LUVs [212].

Fluorescence spectroscopy_is another technique implemented for studies of peptide interaction with model membranes, which provides useful but limited information about the affinity of the peptide for lipid bilayers. It may also shed light on peptide aggregation, location in the membrane and the effects on membrane integrity. When using fluorescence spectroscopy on tryptophan-rich peptides, variations of blue shifts in tryptophan fluorescence is measured, correspond to binding to model membranes. Series of tryptophan-containing β-hairpin peptides have been assessed for their binding to PE/PG and PC/cholesterol-containing vesicles and their binding properties particularly to PE/PG correlates well with their antimicrobial activity [148]. Furthermore, peptides like VRW3 (Ac-C(VR)_3_^D^PG(RV)_3_CW-NH_2_) peptide exhibited stronger binding to PE/PG compared to PC/cholesterol-containing vesicles, while in parallel demonstrating high antibacterial properties and low hemolytic activity [148].

Lipopolysaccharide (LPS) is the endotoxin found on the outer face of the outer membrane of Gram-negative bacteria that helps cell wall stabilization and increases the overall negative charge on the bacterial surface. It is responsible for systemic inflammatory response in mammalians, sometimes leading to septic shock. As a result of simple charge distribution, many cationic antimicrobial peptides are found to exhibit high affinity to LPS. Peptide interaction with LPS is facilitated by displacement of Mg^2+^ which naturally stabilize and cross-bridge adjacent LPS molecules in the outer membrane [123,124]. This interaction further aids the peptides self-promoted uptake granting them access to the inner membrane where they can exert bacterial killing. A contradictory theory suggests that when antimicrobial peptides bind to LPS they tend to form aggregates that are unable to cross the outer membrane and therefore are incapable of killing bacteria [215]. Regardless, when LPS are released as endotoxins, the binding of many cationic antimicrobial peptides will have the potential of neutralizing the toxin, thus they hold promise as new leads for development of improved strategies for clinical treatment of sepsis [216].

### 8.3. Membrane Disruption by Antimicrobial Peptides

Upon membrane binding, antimicrobial peptides contribute to possible alternations of the membrane structure such as thinning, pore formation, altered curvatures, *etc.* This results in overall membrane disruption by lowering the proton gradient (loss of membrane potential) that ultimately stops ATP production and cellular metabolism, leading to cell death [217,218,219,220]. As is the case with many other antimicrobial peptides, membrane permeabilization is a crucial step in the microbicidal activity observed for defensins [18]. The permeability effect has been demonstrated on both mammalian (cell line K562) and bacterial (*E. coli* ML-35) membranes [221,222]. In addition, experiments where defensins were exposed to artificial membranes has shown that channels were formed when negative potential had been applied on the opposite site to a defensin containing solvent [214]. A plethora of evidence supporting various proposed models of specific membrane disruption mechanism exist in the literature. The following section should give a brief overview of some of the acknowledged models.

**Figure 8:**
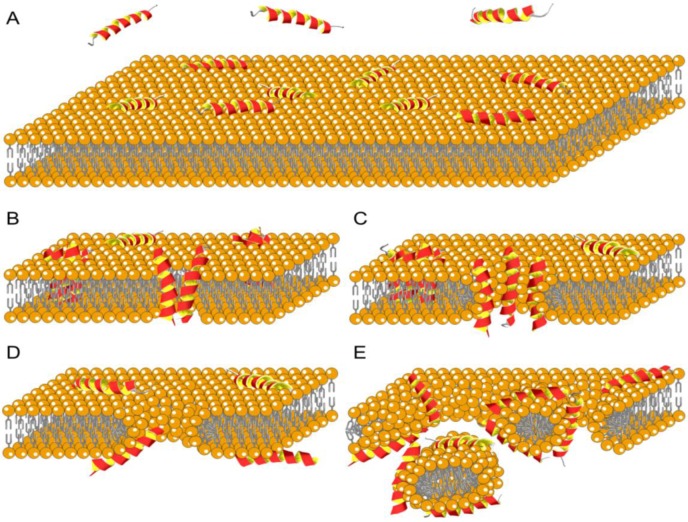
Proposed pathways for peptide interaction and disruption of lipid bilayers. The schematic overview illustrates some of the most well acknowledged pathways. After initial peptide lipid interaction (**A**) different models have been proposed, depending on e.g. peptide type and peptide-lipid ratios. (**B**) The barrel stave model, (**C**) the toroidal pore model, (**D**) disordered toroidal pores and (**E**) the carpet or detergent model. For other suggested models the reader are refereed to [2,232].

One of the first models for pore formation mechanism proposed for antimicrobial peptides is the barrel-stave model [223]. This model has been extensively studied and describes a scenario where after initial binding of the peptides to the membrane, they align perpendicularly to the membrane and aggregate on the surface leading to formation of channels or pores (Figure 8). It has been demonstrated that antimicrobial peptides with defined secondary structures use this mechanism as their hydrophobic parts interact with the lipids of the membrane and the hydrophilic part line the lumen of the pore [105,224]. Alamethicin (Ac-Aib-Pro-Aib-Ala-Aib-Ala-Gln-Aib-Val-Aib-Gly-Leu-Aib-Pro-Val-Aib-Aib-Glu-Gln-Phl) [225] is an antimicrobial peptide that has been extensively studied for its ability to disrupt membranes using this model [226]. Recently, Bobone *et al.* demonstrated and proposed that trichogin GA IV (n-Oct-Aib-Gly-Leu-Aib-Gly-Gly-Leu-Aib-Gly-Ile-Lol) [227] and similarly short peptaibols make use of barrel-stave model to exert their membrane disruption abilities [217]. The two-state model is another perspective on how peptides interact with membranes [211]. Here, the first physical state of interaction is the peptide binding to the membranes (as explained in the earlier section) followed by multi-pore formation at a threshold concentration (or ratio) of peptide to lipid. Furthermore, it has been shown that the lipid composition of the cell membrane is the main determinant for the susceptibility of the cell to an antimicrobial peptide and not the peptides binding affinity [211]. The third interpretation of membrane disruption by antimicrobial peptides supports the toroidal pore model where the peptides cause the membrane to bend inwards so that the pores formed consist of peptides and hydrophilic lipid head groups (Figure 8) [228]. Disordered toroidal pore models have been observed by Sengupta *et al.* using molecular dynamics simulations for melittin and DPPC membranes where peptides were observed to locate near the pore center, while other molecules were positioned close to the pore edge (Figure 8) [229]. The forth model which involves more dynamic disruption of the membrane is the carpet model (Figure 8). Here the peptides bind parallel to the membrane and upon reaching a certain threshold concentration they break the membrane into micelles, resembling a detergent–like mechanism of membrane permeabilization [230,231]. Recently, this model has been proposed for an analogue of PMAP-23 peptide (cathelicidin; RIIDLLWRVRRPQKPKFVTVWV) on its action towards *E. coli* [210]. Killing of the bacteria had only taken place when a complete saturation of the membrane with PMAP-23 molecules occurred, indicating compatible mechanism of membrane destabilization with the carpet model as it has been described using artificial membrane systems [210].

Five other models are suggested by different research groups and those include; interfacial activity [2,232], sinking raft [233], leaky slit [234], lipid clustering [235] and sand in a gearbox [236] models. Reflecting on these conflicting proposed models, one should keep in mind that the challenge to provide universally accepted knowledge regarding precise mechanism of action lies in the different techniques that are applied to this complexity.

### 8.4. Other Mechanisms and Intracellular Antibacterial Targets

Despite the ability to successfully lyse bacterial membranes, there are antimicrobial peptides that can effectively cross the membrane barrier without disrupting it and exhibit their antimicrobial modes of action through intracellular targeting. There is increasing evidence reported in the literature for such antimicrobial peptides and some are described in this section. Human neutrophil peptide 1 (HNP1) and human-β defensin 3 (HBD3) have been reported to bind lipid II, a bacterial cell wall precursor [104,237]. Human β-defensin 3 also affects the electron transport in *S. aureus* [238]. Many antimicrobial peptides have immunomodualtory functions. One example are the human neutrophil peptides 1-3 that when released by tissue of invading granulocytes, trigger secretion of tumor necrosis factor (TNF-α) and interferon-gamma (IFN-γ) from macrophages. This enhances the clearance of bacteria as observed in a murine *in vivo* model [239]. Human β-defensin 3 also activates specialized antigen presenting cells (monocytes, dendritic cells) thus stimulating the adaptive immune system [240]. Buforin II, a 21-amino acid peptide (TRSSRAGLQFPVGRVHRLLRK) [241] with potent broad spectrum antimicrobial activity, is also able to traverse the cell membrane and inhibit cellular function by binding to DNA and RNA of the cells, resulting in rapid cell death [242]. Most recently, it has been documented that indolicidin binds double stranded DNA, thereby inhibiting DNA replication and transcription [39]. Another non-lytic antimicrobial peptides is the small proline-rich apidaecin peptide that kills bacteria by binding to a cytoplasmic target, most likely DnaK [243].

## 9. Mechanism of Bacterial Resistance towards Antimicrobial Peptides

Antibiotic treatments to fight various infections have greatly contributed to increased human life expectancy throughout the years. However, only a year after the discovery and the commercial use of penicillin, resistant strains were already isolated due to high selection pressure and rapid resistance development. The same applies to other antibiotics to which many bacteria have developed resistance, thus there are many infections today that lack treatment options. Antimicrobial peptides hold potential for being part of the novel anti-infective strategies. The majority of the peptides act on the microbial membrane and not on specific intracellular targets, with a mechanism less prone to development of resistance. However, to resist the action of antimicrobial peptides, bacteria have evolved various strategies. These include, proteolytic degradation, shielding bacterial cell surface, surface modification of membrane structure, active efflux and down-regulation of antimicrobial peptide expression [244]. Proteolytic degradation has been reported for LL-37 and β-defensin 2, by proteases from *P. mirabilis* [245] in addition to many proteases secreted by bacteria that cleave peptides after specific amino acid residues. In Gram-negative bacteria, specifically, many of the proteases that inactivate antimicrobial peptides are found at the outer membrane [246]. Extracellular structures such as capsule polysaccharides, fimbriae, exopolysaccharides and *O*-polysaccharide of LPS aid bacteria by binding antimicrobial peptides and thereby reducing the amount of peptide reaching the bacterial membrane [247,248,249,250,251]. Addition of positively charged groups or removal of phosphate group of lipid A, thereby neutralizing and decreasing the negative charge that attracts cationic antimicrobial peptides, respectively, has been reported as a strategy to resist the action of polymyxin B (Figure 6) [252,253]. Decreased overall negative charge has further been reported as a resistance mechanism for cationic peptides and related mimetics [254,255,256,257]. Gram-positive bacteria also resist the action of antimicrobial peptides also by enzymatic degradation by extracellular proteases [258] and cell wall and membrane modifications. The latter is prevalent by deacetylation of *N*-acetylglucosamine or *O*-acetylation of *N*-acetylmuramyl residues in the cell wall and alternation of charge by d-analynation of the lipoteichoic acids [259]. One example is d-alanylation of anionic lipoteichoic acids in group B *Streptococcus* via increasing the cell wall density and thereby reducing the penetration of cationic antimicrobial peptides [260]. Another way to resist antimicrobial peptide activity is to pump these antimicrobials out of the bacterial cytoplasm. Bacteria use import and export pumps for the movement of different molecules across their membrane. By using bacterial strains with deleted or inactive pumps, it has been demonstrated that these mutant strains are significantly more susceptibility of antimicrobial peptide killing [261,262]. In parallel to regulation of its own defense strategies, several bacteria also produce toxins that affect host recognition of bacteria. e.g., The exotoxins of *Vibrio cholerae* and enterotoxigenic *E. coli* have both been reported to be involved in down-regulation of antimicrobial peptides, *i.e.*, LL-37 and hBD1, expression by host cells, although the precise mechanism behind this still is unknown [263]. In summary, bacteria as any living organisms, will always adjust to a selective pressure through various modes of resistance. The chemical evolution of antimicrobial peptides has proven to counteract some of the resistance mechanisms through various structural modifications and thus keeps antimicrobial peptides in the game as potential antimicrobial alternatives to the conventional antibiotics.

## 10. Peptidomimetics for Antimicrobial Research

**Figure 9:**
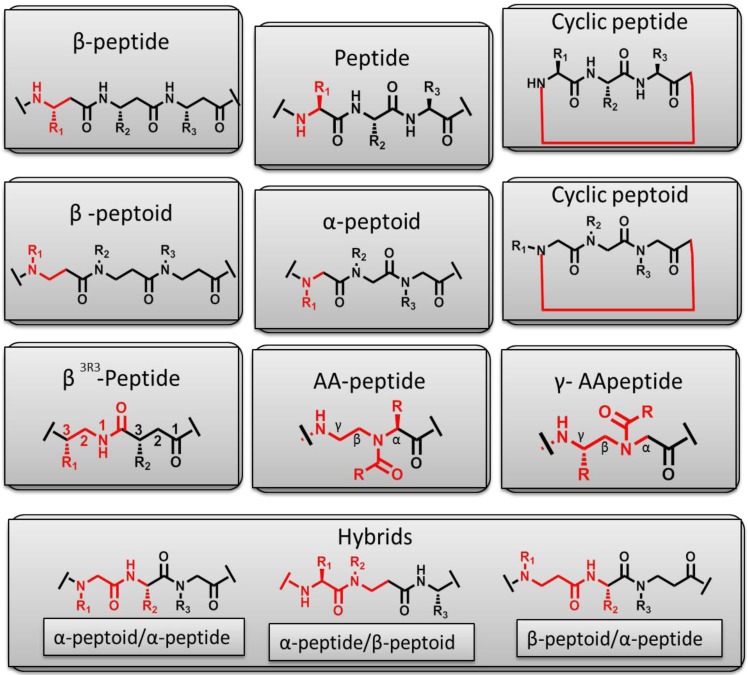
Current peptidomimetic structures with potent antimicrobial activity. The schematic illustrates the different backbone compositions of a variety of peptide and peptidomimetic structures that has been proven to possess antibacterial properties.

In the continuous research for novel antimicrobial drugs, antimicrobial peptides serve as a pharmacophore for structural optimization. In order to retain the activity and selectivity of antimicrobial peptides, while improving bioavailability, metabolic stability and immunogenicity, researchers has challenged the peptide structures with various diverse modifications. Any compound that is able to imitate the structural properties and/or biological activities of a peptide is referred to as a peptidomimetic. Modifications of peptide structures in antimicrobial research involve backbone and/or side chain modifications. Some examples include incorporation of unnatural amino acids (e.g., d*-*amino acids), β-peptides, peptoids (*N-*substituted glycines), hybrid peptide-peptidomimetic structures, lipidation *etc.* (Figure 9). This section highlights some of the most common peptidomimetic strategies in antimicrobial drug research and development.

### 10.1. Unnatural Amino Acid Sequences

Some of the different strategies to modify and improve the antimicrobial properties of antimicrobial peptides include substitution of the natural L-amino acids with unnatural analogs. However, one should keep in mind that the unnatural amino acids are not always synthetic and thus several different types are found in nature. One example is the natural derivatives of L*-*proline that are found in leucinostatine, a peptide antibiotic produced by an endophytic fungus of European yew (*Taxus baccata*), which has demonstrated broad spectrum antimicrobial activities [264]. Structural modification of the side chains of arginine and lysine has also been investigated in a synthetic library of tritpticin (VRRFPWWWPFLRR)-derived peptides. By methylation of the side chain and also alternation of the length, it was demonstrated that the antimicrobial activity of the peptides could be slightly improved while the hemolytic activity was decreased when compared to the parent tritpticin peptide [265].

Another very common modification strategy used to improve on traditional antimicrobial peptides has been to change from l- to d-amino acids. d*-*Amino acids are very rare in Nature and incorporation of the D*-*amino acids changes the side chain and the backbone properties of the peptide. In addition, *retro-inverso* peptides have also been introduced, representing a reversed peptide sequences from *N-* to *C-*terminus exchanging l*-*amino acids to d*-*amino acids [266]. One of the first examples of a peptidomimetic is the all-d*-*magainin peptide, however this peptide failed to show any significant improvement of the antimicrobial activity when compared to the all-l*-*enantiomer. However, all-d*-*magainin demonstrated high resistance to proteolysis and exhibited no hemolytic activity, two properties which are considered valuable for therapeutic applications [267].

### 10.2. Addition of Lipid Moieties

Natural lipopeptides are well characterized in the literature as promising antimicrobial compounds, often produced by different bacterial strains to give an advantage over other closely related strains. This family of bacterial compounds generally includes cationic, cyclic compounds, such as polymyxins (B and E), daptomycin, lipopeptaibol and others (Figure 6) [268]. Consequently, addition of an aliphatic chain to the N*-*terminus of an active antimicrobial peptide can be a strategy to improve the peptides overall antimicrobial properties [269,270]. Cudic *et al.* have actively investigated novel lipopeptides by incorporating synthetic analogues to structures of known antimicrobials [145]. To exemplify, they recently designed a novel cyclic lipopeptides (cyclo-[D-Ala-(12-guanidino-dodecanoyl)Thr-D-Val-Val-DaThr-D-Asn]) (Figure 6) derived from fusaricidin, which proved to inhibit both growth of *S. aureus* biofilms *in vitro* and proliferation of *S. aureus in vivo* [271]. By using combinations of hydrocarbon tails and other hydrophobic moieties in conjugation with polylysine and lysine analogues, Ahn *et al.* [272] have demonstrated successful design of very simple antimicrobial peptides, with no detectable hemolytic activity [272]. Though their precise mode of action still needs to be characterized, they present convincing data demonstrating that the peptides work through different mechanisms than the classical membrane targeting peptides like melittin [272].

### 10.3. β-Peptidomimetics

The modification achieved in β-peptidomimetics involves a change in the backbone of the natural peptide structures without changing the side chain chemistry, *i.e.*, addition of one, two or three carbons along the peptide chain (Figure 9). A small library of highly potent, short β-peptidomimetics has been synthesized by Hansen *et al.* [273]. The library was designed to mimic the minimal pharmacophores model for ultra-short peptides with activity against *S. aureus.* The β-peptidomimetics consisted of different lipophilic β^2,2^-amino acid coupled to a *C*-terminal amidated *L-*Arg residue. The most potent peptidomimetic from this library displayed MIC values of 2.7–7.2 µM against *S. aureus*, methicillin-resistant *S. aureus* and *S. epidermidis,* as well as and *E. coli*, thus presenting one promising class of antimicrobial agents with improved enzymatic stability and a low cost production [273]. Another interesting novel class of active peptidomimetics has been introduced by Mosca *et al.* [274]. This class presents amphiphilic cationic β^3R3^-peptides. These peptides demonstrate high selectivity with low cytotoxicity profile and thus illustrate a class of antimicrobial candidates with high therapeutic index with potential for further development.

### 10.4. Peptoids

Peptoids are oligomers of *N*-substituted glycines. They comprise a new class of unnatural compounds that mimic peptide structures by relocating the side chain from the α-carbon to the nitrogen (Figure 9). Structurally, the change in the amide bond corresponds to a loss of backbone chirality which can be compensated for by introducing chiral side chains that allow peptoids to fold into stable secondary structures [275,276,277]. Two of the most appreciated advantages with the backbone modification in peptoids are that side chains appended at the nitrogens render them less prone to enzymatic and proteolytic degradation and they are often more membrane permeable than peptides [278]. In this manner, Bang *et al.* used the peptoid conversion strategy of the novel tryptophan-rich model peptide to increase its protease stability while retaining its biological activity [279]. For more than 15 years, the research in peptoid synthesis and application increased dramatically due to their potential as compounds with broad antimicrobial activity profiles [280]. Their synthesis comprises a two steps process in which various side chains of commercial availability are incorporated and this versatility enables peptoids to hold status as promising structures for antimicrobial drug development. The careful work by the Barron group provides important information on several helical, cationic, facially amphipathic peptoid mimics of magainin-2 amide. Certain analogues exhibited potent antimicrobial activities against both Gram-negative and Gram-positive bacteria with low toxicity against human red blood cells [281]. In addition, application of peptoids as effective antimicrobials against *Mycobacterium tuberculosis* and inhibitors of biofilm formation by *P. aeruginosa* has been reported [282,283]. High synergistic interactions between nine antimicrobial peptides and peptoids have been further demonstrated suggesting that these two classes of antimicrobials are functionally and mechanistically analogous [284]. Recently another study demonstrated synthesis of peptoids that mimic the structure of antimicrobial peptides while retaining good potency against broad spectrum bacterial and low toxicity against human cells [285]. Overall, the important and conserved properties of antimicrobial peptides are also seen in peptoids and as such provide adequate information for future design of potent antimicrobials.

### 10.5. Cyclic Peptoids

Another structural challenge that has been overcome by Kirshenbaum *et al.* is cyclization of peptoids via a head to tail macrocyclization reaction [286]. Soon after an efficient synthetic approach was established, a comparison of linear and cyclic peptoids (6-10 residues) with potent antimicrobial activity demonstrated that the cyclic counterparts exhibited higher antimicrobial activities with no significant lysis of human erythrocytes [287]. New studies have shown that cyclic peptoids can damage methicillin-resistant *S. aureus* membranes trough pore formation mechanism with low MIC and low hemolytic activity [288]. Furthermore, these study challenge many researches in the peptoid field to embrace the cyclization awareness in developing peptoids as candidates for antimicrobial drugs.

### 10.6. Hybrids

Another attractive approach in peptidomimetics synthesis is merging structures of native peptides and their mimics which results in hybrid structures. For example, substituting arginine/leucine residues in apidaecin Ib with peptoid residues has shown to circumvent the resistance problem without any significant cytotoxic effects, however this has led to reduced antimicrobial activity at specific positional substitutions due to the inability of the novel peptide-peptoid hybrid to translocate into bacterial cells [289]. Research on piscidin 1, a novel cytotoxic peptide with cationic α-helical structure, provided support of an increased antimicrobial activity and lower cytotoxicity in mammalian cells by substitution of Pro_8_ with lysine mimicking peptoid residues (*N*lys). This modification provided structural flexibility of the novel compound that exhibited better membrane permeability in bacteria conferring better overall selectivity [290]. The study of antimicrobial profiles of peptoid hybrids draws on research conducted by Franzyk *et al.* which described the first generation of oligomers consisting of alternating repeats of α-amino acids and β-peptoid residues. Such structures showed stability toward proteolysis, and exhibited high antimicrobial and no hemolytic activities. Additionally, more in depth evaluation of the synthesized library of hybrids challenged the importance of the length, choice of cationic side chain, presence of chiral side chains as well as lipophilicity on both the antimicrobial and hemolytic activities observed by this library of compounds [291,292,293,294]. Hansen *et al.* demonstrated synthesis of 20 novel lysine-peptoid hybrids designed on the basis of an active parent structure [*N-*(1-naphahalenemethyl)glycyl] - [*N-*(4-methylbenzyl)glycyl] - [*N*-(1-naphthalenemethyl)glycyl]-*N*-(butyl) -glycine amide 1, with antimicrobial properties against both Gram-negative and Gram-positive bacteria [295]. Recently, more profound information on HDM-4 peptidomimetic, composed of repeating units of lysine and *N*Phe (benzylamine), was demonstrated. This peptidomimetic exhibits low toxicity against mammalian cells and kills Gram-negative bacteria by membrane disruption. DNA binding, induction of chemokine production in immune cells and inhibition of LPS induced pro-inflammatory response has also been reported for this peptidomimetic [296]. In parallel to these observations, similar behaviour for DNA binding of peptide-peptoid hybrid has been established for lysine-peptoid hybrid, LP5. LP5 inhibited the growth of *S. aureus in vitro* by binding to DNA and inhibiting macromolecular synthesis. In addition it inhibited DNA gyrase and topoisomerase IV causing SOS response [297]. In summary, synthetic analogues and mimetics of antimicrobial peptides are being actively developed and studied to improve the antimicrobial and pharmacokinetic properties and lower the cost of production.

### 10.7. AApeptides

Another class of peptidomimetics with broad spectrum antimicrobial activity are AApeptides. This class of antimicrobials have been developed by Cai *et al.* [298] based on the chiral peptide nucleic acid backbone (Figure 9). Various structural modifications such as lipidation and cyclization have been illustrated for AApeptides and these new structures have been shown to be active against a range of community-acquired multidrug resistant pathogens [299,300,301].

## 11. Towards the Design of Novel Antimicrobial Peptides

The main goal of all researchers in the field of antimicrobial peptides is to contribute to the antimicrobial peptide range with novel strategies for the development of pharmacologically relevant antimicrobial peptide structures. The design of *de novo* structures is not always evident and it demands extensive experimental work. To meet this challenge and aid the discovery of *de novo* peptides, various computer-based strategies have been employed. Most involve building quantitative structure-activity relationship parameters for computer aided design of antimicrobial peptides. The general idea is to combine chemical sequence and structure of antimicrobial peptides, described with physiochemical parameters (descriptors) and correlate them with their respective biological activities using mathematical models [304,305]. One of the mathematical models used for prediction of novel polypeptide sequences with potentially improved biological activities is the Designer algorithm [306,307]. Using the Designer algorithm the most recent work of Ilic *et al.* reports on a series of peptide sequences with high antibacterial activity against Gram-negative bacteria (0.5–4 µM) and low hemolytic properties (HC_50_ > 400 µM) [308]. Fjell *et al.* have also created a software system that could identify peptides with antibacterial activity with up to 94% accuracy [309,310]. As a new method that further improved the identification of novel antimicrobials, they describe the programming method of genetic algorithms, improving on the number of active peptides within a semi-random library [311]. In summary, this kind of models can be useful for prediction of antimicrobial peptides with potentially higher biological activities; however the challenge to develop a robust model still prevails.

## 12. Concluding Remarks

There is increased evidence of emergence of bacteria resistant to conventional antibiotics illustrating the importance of research on antimicrobial drug development. Antimicrobial peptides and their synthetic derivatives hold vast potential in the development of novel antimicrobial drugs due to their (1) high biological activities; (2) low cost of production when compared with that of proteins and antibodies; (3) ease of structural modifications and stability improvements; (4) promising pharmacokinetic profiles; (5) degradation that leads to amino acids which are less toxic for the organism resulting in low immunogenicity and (6) good organ penetration.

The variety of structure-activity relationship studies for antimicrobial peptides demonstrate that the observed antibacterial activity is usually the outcome of multiple factors such as; secondary structures, amphipathicity, charge, length and hydrophobicity. The sequence composition together with the importance of specific residues and their contribution to the optimal therapeutic profiles of the peptides reveal important scaffolds for design of novel compounds to successfully combat and eliminate infectious diseases in the future.
